# Seizure Prophylaxis After Intracerebral Hemorrhage in a Patient With Psychiatric Illness: A Case Report

**DOI:** 10.7759/cureus.94185

**Published:** 2025-10-09

**Authors:** Daniel Newman, Konstantinos Mouskas, Risit Datta, Dylan Miller, Cristina Suarez Chiriboga, Roxana Lazarescu

**Affiliations:** 1 Internal Medicine, Touro College of Osteopathic Medicine, New York, USA; 2 Neurology, Wyckoff Heights Medical Center, New York, USA; 3 Internal Medicine, Wyckoff Heights Medical Center, New York, USA

**Keywords:** antiseizure medication, bipolar disorder, epilepsy, intracerebral hemorrhage, levetiracetam, oxcarbazepine, schizophrenia

## Abstract

Psychiatric comorbidities are highly prevalent in patients with epilepsy. This can complicate the management of seizures, as antiseizure medications (ASMs) can interact with psychotropic drugs or worsen psychiatric symptoms. We report the case of a 70-year-old woman with schizophrenia and bipolar disorder who presented with acute confusion and was found to have an elevated serum lithium level and an acute right temporal lobe intraparenchymal hemorrhage. Levetiracetam was initiated for seizure prophylaxis but was discontinued due to concern for psychiatric side effects. Oxcarbazepine was started and provided seizure protection with better psychiatric tolerability. This case illustrates the unique challenges of seizure prophylaxis in patients with psychiatric comorbidities.

## Introduction

People with psychiatric disorders have a higher risk of experiencing seizures and developing epilepsy. Further, psychiatric conditions, such as depression, anxiety, bipolar disorder, schizophrenia, and substance use disorders, are more common in people with epilepsy. An estimated 35% of patients with epilepsy experience psychiatric disorders, which is two to three times higher than in the general population [1]. Specific conditions, including major depressive disorder and generalized anxiety disorder, occur in roughly one-third of patients, bipolar disorder in about 4.5%, and psychotic disorders in about 6% [2]. Substance use disorders add more issues, contributing to nonadherence, drug interactions, and increased morbidity.

Antiseizure medications (ASMs) can affect psychiatric care by causing mood and behavioral side effects and by interacting with psychotropic medications, which in patients with preexisting psychiatric illness, may exacerbate underlying symptoms or diminish treatment effectiveness. Choosing the right ASM, therefore, requires balancing the need for seizure control to minimize psychiatric side effects and preserve psychiatric stability [3]. These overlapping interactions highlight the importance of individualized treatment planning and careful coordination between neurology and psychiatry when selecting an ASM for patients with psychiatric conditions.

We present a case of an older woman with severe psychiatric comorbidities who required seizure prophylaxis after an intracerebral hemorrhage (ICH) to illustrate this difficult situation. This case highlights the clinical challenge of selecting an ASM that preserves psychiatric stability while preventing seizures and avoiding harmful drug interactions. The objectives of this report are to review the challenges of ASM use in patients with preexisting psychiatric conditions and to examine the unique challenges of inpatient seizure prophylaxis in these patients.

## Case presentation

A 70-year-old woman with a history of schizophrenia and bipolar disorder for about 20 years presented to the emergency department due to declining mentation. On initial assessment, she was alert but oriented only to self, with fragmented speech, a flat affect, apraxia, and a small hand tremor, as well as hallucinations that someone was trying to kill her. Given her extensive psychiatric history and medication regimen, including lithium (450 mg once a day), haloperidol (5 mg twice a day), benztropine (0.5 mg twice a day), clonazepam (2 mg twice a day), and zolpidem (10 mg once a day), there was immediate concern for medication toxicity. This suspicion was supported by her initial laboratory evaluation, which revealed an elevated serum lithium level (1.34 mmol/L). Other laboratory findings include an elevated BUN of 25 mg/dL, a decreased eGFR (48 mL/min/1.73 m²), and an upper borderline creatinine level (1.21 mg/dL). Lithium and benztropine were discontinued due to suspected toxicity and renal impairment, while clonazepam was stopped due to concerns for sedation and fall risk.

**Figure 1:**
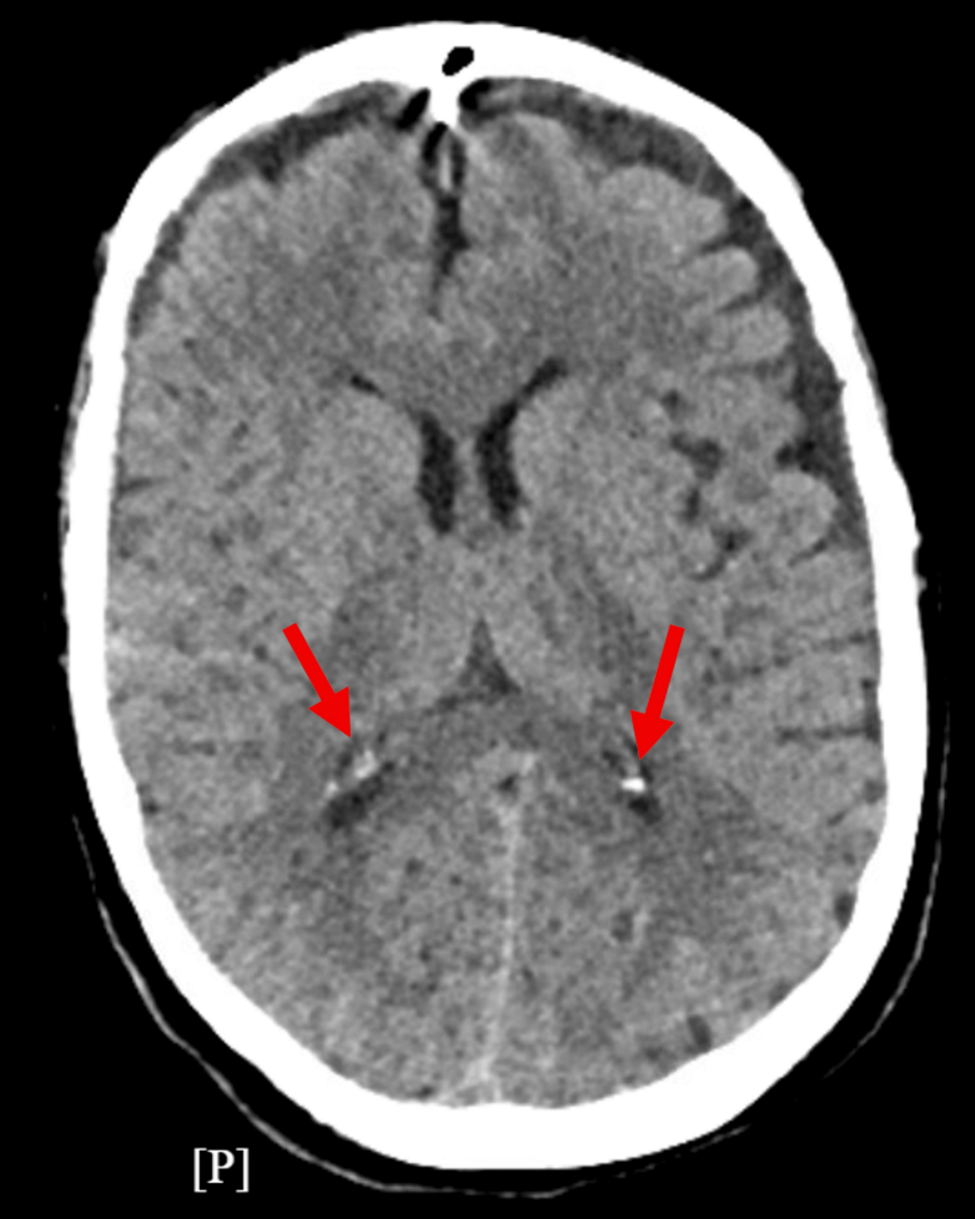
Axial non-contrast head CT demonstrates hyperdense foci concerning an acute ICH. The lobar distribution involving the cortex increases the risk of seizure activity. CT: computed tomography, ICH: intracerebral hemorrhage

A head CT demonstrated cortical volume loss, bilateral small frontal subdural hygromas, and small hyperdense components concerning for possible acute hemorrhage (Figure 1). For behavioral control, quetiapine was initiated, and she was also started empirically on dexamethasone for the CT findings.

**Figure 2:**
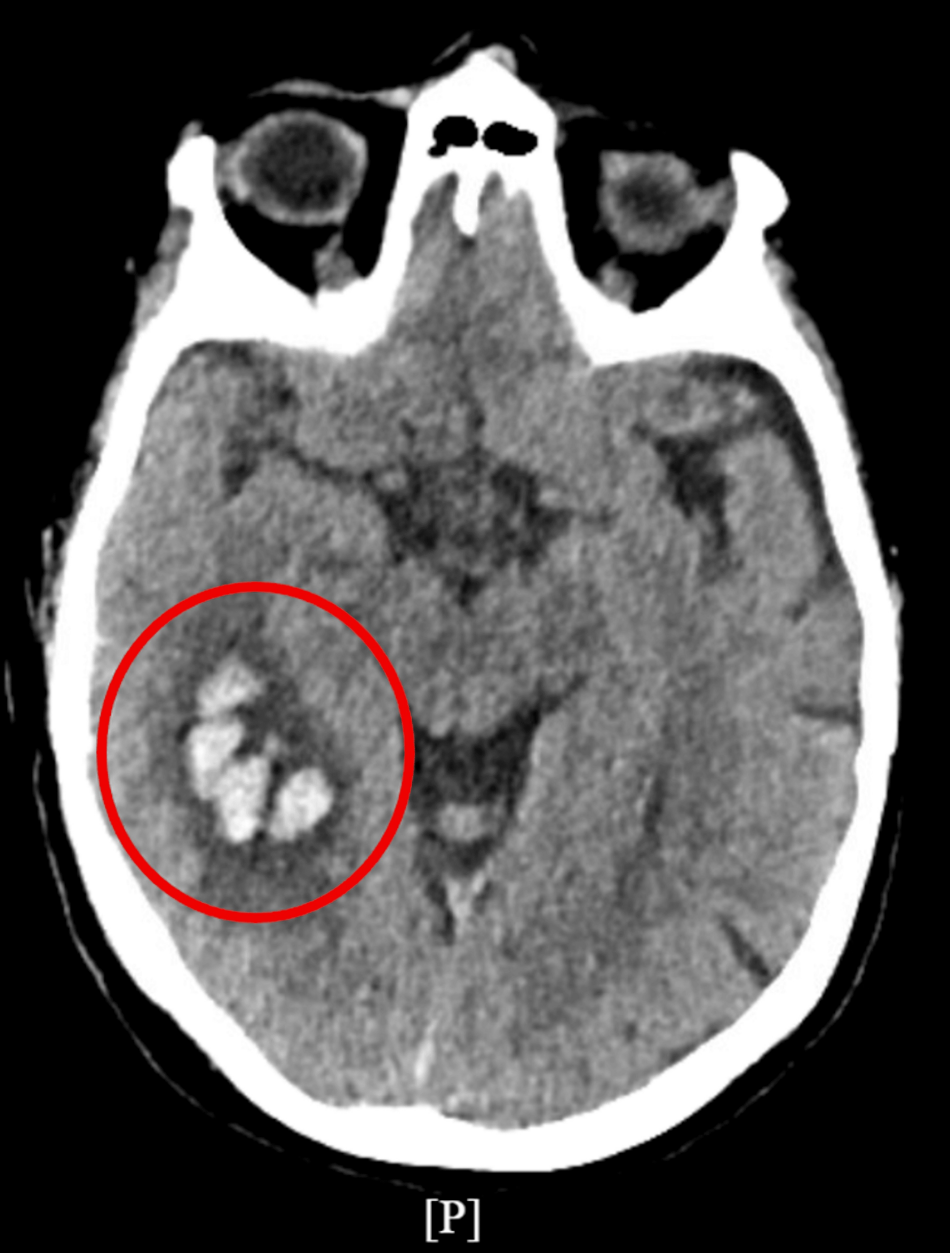
Repeat axial non-contrast head CT demonstrating an acute right posterior temporal–occipital intraparenchymal hemorrhage measuring 4.1 × 2.7 cm, associated with increased seizure risk. CT: computed tomography

Despite these interventions, a repeat non-contrast head CT revealed new pathology of an acute intraparenchymal hemorrhage in the right posterior temporal/occipital lobe measuring 4.1 × 2.7 cm, with surrounding vasogenic edema but no midline shift. The bilateral subdural hygromas with possible small punctate hemorrhages in the left frontal and parietal lobes remained stable (Figure 2). Given the new hemorrhage, she was transferred to the medical ICU for closer neuro-monitoring. The etiology was thought to be non-traumatic, with a differential diagnosis including cerebral amyloid angiopathy, hemorrhagic transformation of an ischemic stroke, or, less likely, an underlying mass. Levetiracetam was initiated for seizure prophylaxis, and a subsequent CT scan demonstrated stability of the hemorrhage.

**Figure 3:**
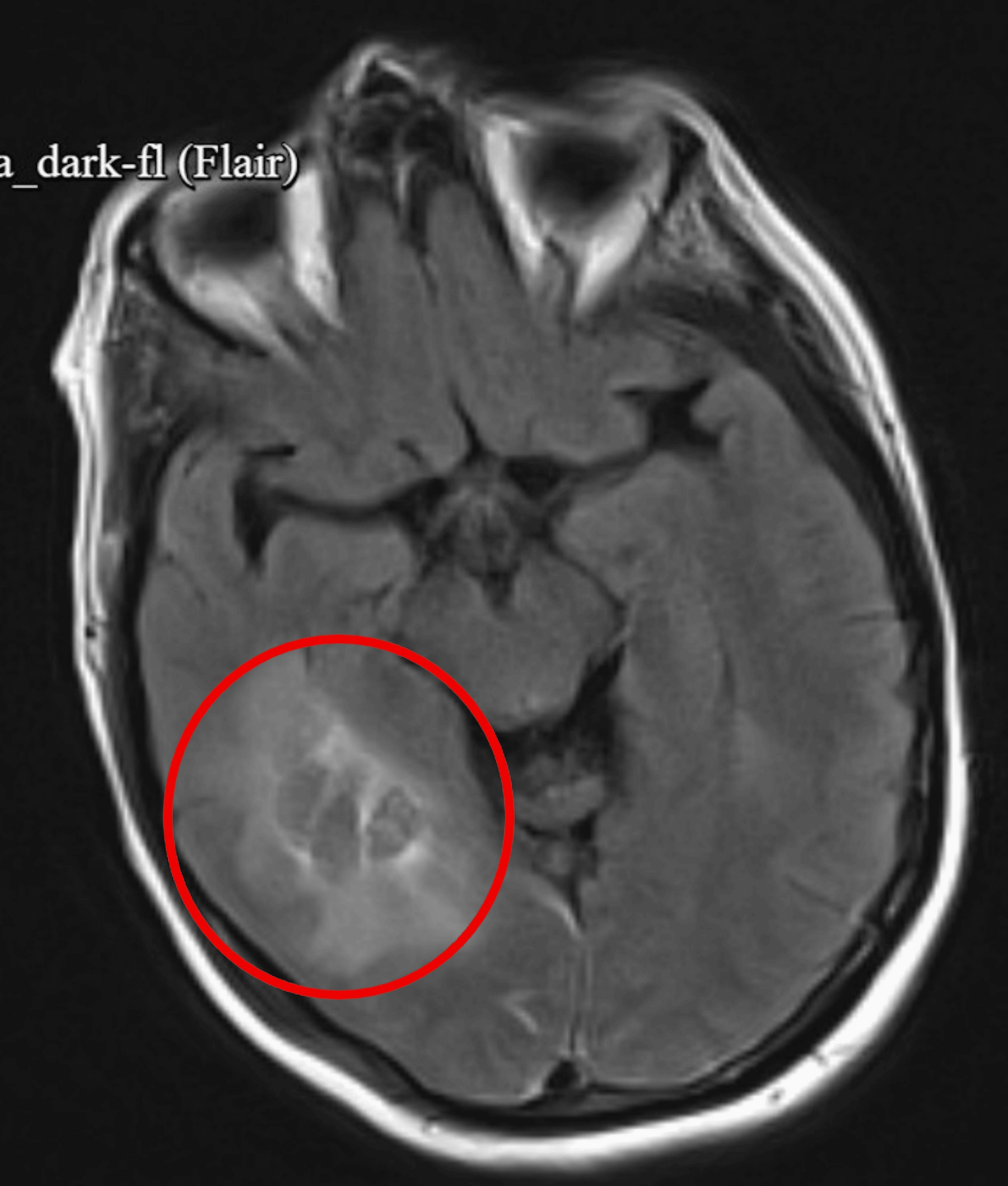
Axial FLAIR brain MRI demonstrates a subacute right temporal intraparenchymal hemorrhage. FLAIR: fluid-attenuated inversion recovery, MRI: magnetic resonance imaging

**Figure 4:**
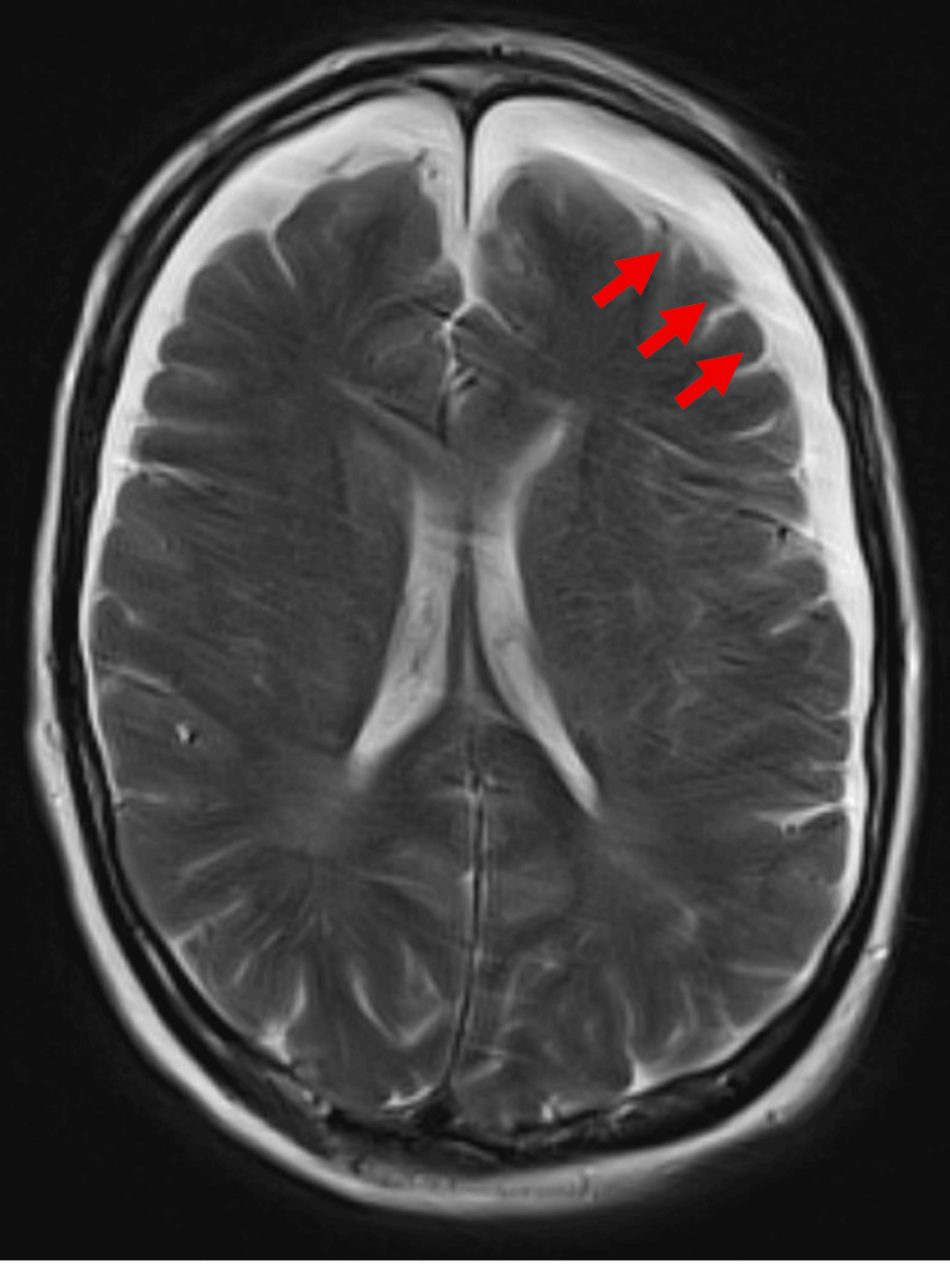
Axial T2 brain MRI showing an acute-on-chronic left frontal subdural hematoma. MRI: magnetic resonance imaging

In the days that followed, her condition continued to stabilize. Brain MRI revealed a subacute right temporal intraparenchymal hemorrhage with significant vasogenic edema, as well as an acute-on-chronic left frontal subdural hematoma, mild generalized cerebral atrophy, and chronic small vessel ischemic changes (Figures 3-4). At this point, her persistent encephalopathy raised concern for levetiracetam-related sedation. In response, neurology transitioned her to oxcarbazepine for long-term seizure prophylaxis. Additionally, psychiatry reduced quetiapine and advised against restarting lithium, benztropine, or benzodiazepines.

Over the course of her hospitalization, the patient’s mental status gradually improved. By hospital day 11, she was more alert, eating with assistance, and beginning to interact with her environment. With her condition medically stabilized, she was ultimately deemed appropriate for discharge to a skilled nursing facility.

## Discussion

The management of seizures in patients with preexisting psychiatric conditions is uniquely challenging, as the choice of ASM must balance efficacy with psychiatric safety. While the primary goal of ASM use is to prevent seizures and minimize neurological morbidity, psychiatric side effects and drug-drug interactions can significantly alter both adherence and overall quality of life. The following discussion highlights these considerations through the lens of our patient’s clinical course, emphasizing the importance of tailoring ASM therapy to psychiatric comorbidities, polypharmacy, and long-term outcomes.

In this context, the use of levetiracetam, which was initially prescribed for our patient, exemplifies both the strengths and limitations of standard practice. Levetiracetam is typically the first choice ASM for seizure prophylaxis after ICH due to its efficacy and minimal drug-drug interactions. However, it is also among the ASMs most strongly linked to behavioral and psychiatric side effects [4]. In our patient, who was already experiencing psychotic symptoms and delirium, continuation of levetiracetam posed a substantial risk of worsening agitation, underscoring the need to consider psychiatric history in ASM selection. Given this, switching to oxcarbazepine was clinically justified. While there are side effects such as hyponatremia and sedation, oxcarbazepine has a better psychiatric profile and is also considered to have mood-stabilizing properties [5]. Moreover, several other ASMs are commonly considered in patients with psychiatric illness, each with different benefits and drawbacks, which underscores the need for individualized selection based on patient-specific factors.

Since our patient had a long-standing history of schizophrenia and bipolar disorder, they had an extensive home medication list. First, the combination of lithium and haloperidol has been linked to serious neurotoxic reactions such as confusion, tremor, rigidity, and extrapyramidal symptoms [6]. Further, to counteract haloperidol-induced dystonia, benztropine was added, which carries its own risk of anticholinergic side effects of dry mouth, constipation, and urinary retention [7]. The risks of lithium toxicity are particularly pronounced in older adults, where reduced renal clearance further narrows the therapeutic window [8]. In the setting of the patient’s intracranial hemorrhage, her critical illness and unstable volume status placed her at greater risk for kidney injury. This vulnerability, combined with lithium’s renal clearance, likely increased her susceptibility to acute kidney injury and amplified its toxic effects.

While seizure control is a complex issue in this population overall, this case also highlights the unique challenges of using seizure prophylaxis after ICH. Current guidelines from the Neurocritical Care Society advise against routine long-term use, as evidence does not support reductions in seizures [9]. Additionally, the use of seizure prophylaxis may even lead to worse functional outcomes and a greater risk of ASM side effects. Instead, it is recommended for short courses in patients at the highest risk for early seizures, such as those with lobar hemorrhage or cortical involvement. Further, the guidelines state that seizure prophylaxis should not be used in patients who have suffered from a non-traumatic ICH, such as in our current case [10]. In clinical practice, levetiracetam is the first choice for seizure prophylaxis after ICH due to its improved safety, reduced drug-drug interactions, and lower risk of long-term cognitive impairment [11]. However, its advantages are not seen in patients with significant psychiatric comorbidity, as the behavioral side effects can worsen psychiatric conditions, such as increasing agitation, causing mood changes, and triggering psychosis. This risk was especially relevant in our patient, who presented with schizophrenia, bipolar disorder, and altered mental status, making continuation of levetiracetam dangerous.

The decision to transition to oxcarbazepine, though atypical for post‑ICH seizure prophylaxis, reflected an individualized approach. Oxcarbazepine is better for patients with preexisting psychiatric conditions and may have mood‑stabilizing effects. This case illustrates that the choice of ASM in ICH should not be determined solely by seizure risk but must also consider psychiatric comorbidities, medication tolerability, and overall quality of life, which substantially influence long-term outcomes. Neurology and psychiatry must coordinate treatment together to minimize polypharmacy and support patient safety. Importantly, patients with preexisting psychiatric illness remain underrepresented in clinical trials, and more research is needed to guide seizure prophylaxis strategies in this population.

## Conclusions

This case highlights the challenges of managing seizure risk in psychiatric patients with ICH. These patients are at an increased risk for behavioral side effects and polypharmacy due to their illness and medication profile. There are several broader implications of this case that physicians should consider. First, the ASM choice in patients with psychiatric illness requires individualized assessment that accounts for psychiatric history, medical comorbidities, and current psychotropic medications. Second, future research on ASM choice should specifically include patients with psychiatric illness to develop evidence-based guidelines to improve outcomes. Overall, this is a critical case study demonstrating the importance of careful clinical decision-making in psychiatric patients with ICH.
